# Delayed enhancement and myocardial velocity mapping CMR reveal differences in regional left ventricular function with varying levels of scar

**DOI:** 10.1186/1532-429X-15-S1-P81

**Published:** 2013-01-30

**Authors:** Amita Goyal, Darshit Thakrar, James Carr, Jeremy Collins, Michael Markl, Jacob Fluckiger

**Affiliations:** 1 Northwestern University Feinberg University, Chicago, IL, USA; 2 Northwestern Memorial Hospital, Chicago, IL, USA; 3 Ann and Robert H Lurie Childrens Hospital of Chicago, Chicago, IL, USA

## Background

Delayed enhancement (DE) CMR is the gold standard for detecting irreversibly damaged myocardial tissue (scar) . Yet, direct impact of scar on regional systolic and diastolic left ventricular (LV) function is not well understood. Standard tools for LV velocities (Tissue Doppler Imaging) are limited by poor reproducibility and incomplete assessment of all regions and motion directions. Myocardial CMR velocity mapping (MVM) is reproducible, non-invasive, and allows direct measure of myocardial velocities of all LV motion components in all regions. Here, we analyze effects of LV scar burden on myocardial motion.

## Methods

CMR (1.5T Avanto, Siemens) with DE-CMR (inversion recovery PSIR) and MVM in 49 patients (age=54±16 years) with cardiomyopathy (n=16), normal myocardium (n=10), inflammatory disease (n=4) ischemic heart disease (n=3), heart failure (n=3), and other (n=13) were acquired in basal, mid-ventricular, and apical short axis orientation during breath hold. DE-CMR: A radiologist assessed whether scar was present in segments from the AHA 16-segment model and classified based on number of segments with scar (no scar=0, low scar=1-2, high scar=3+). MVM: Consisted of black-blood prepared CINE phase-contrast sequence with 3-directional velocity encoding (venc=25cm/s, temporal resolution 25ms; spatial resolution=2.9x2.4mm, slice thickness=8mm, k-t parallel imaging with Rnet=3.6). Data analysis included manual segmentation of LV contours and conversion of measured velocities into radial, rotational, and long-axis velocities. Systolic and diastolic radial and long-axis velocities were derived and mapped on the AHA 16-segment model. Global (average all segments) radial and long-axis velocities were analyzed using t-test.

## Results

**Figure 1:**
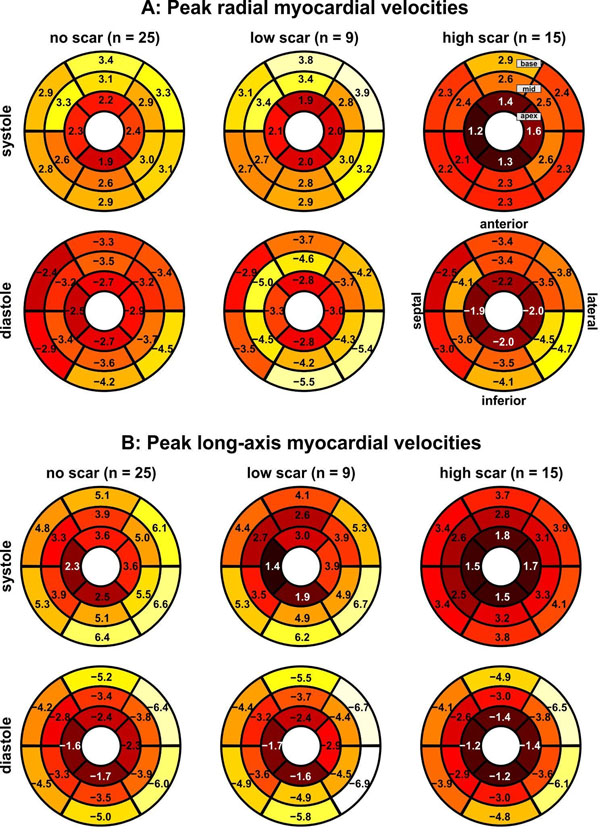
Systolic and diastolic left ventricular function in patients without evidence of scar on delayed enhancement MRI (left, no scar, n=25) compared to a group of n=9 patients with low scar burden (evidence of scar –n 1-2 segments) and n=15 patients with high scar burden (evidence of scar in 3 or more segments). The individual plots show the distribution of peak systolic and diastolic radial (A) and long-axis (B) myocardial velocities in the AHA 16-segment model.

Patients with high scar showed reduced regional peak velocities compared to patients with no and low scar (figure 1). Systolic and diastolic radial velocities (figure 1A) for patients with high scar were reduced in all segments compared to no and low scar groups. Consistently, global peak radial velocities (table 1) were notably reduced for both systole (p<0.0001) and diastole (p=0.003). Also, patients with high and low scar showed significantly different systolic global radial velocities. Changes in regional long-axis velocities (figure 1B) were less evident and clearest in systolic velocities (notably reduced global systolic peak velocities in patients with high scar vs. no scar (p=0.012, table 1)).

**Table 1:** Global redial and long-axis (± standard deviation) velocities over all segments for each of the patient cohorts. P-values less than 0.05 denote significant differences between cohorts.

LV velocities	No Scar [cm/s]	Low Scar [cm/s]	High Scar [cm/s]	p-value (No vs. Low)	p-value (No vs. High)	p-value (Low vs. High)
Radial - systole	2.79±1.09	2.85±0.99	2.16±1.20	0.54	<0.0001	<0.0001
Radial - diastole	3.25±1.37	3.96±1.73	3.27±1.87	<0.0001	0.92	0.0003
Long-axis - systole	4.56±2.99	4.04±2.82	2.89±2.20	0.53	0.012	0.11
Long-axis - diastole	3.75±2.21	4.18±2.62	3.40±2.26	0.41	0.34	0.19

## Conclusions

DE-CMR and MVM, measuring scar burden and regional myocardial velocities, were co-registered to detect impaired regional myocardial structure and function. Segmental analysis showed significant decreases in mean velocities in patients with high scar indicating a direct relationship between structural damage to the LV and impaired myocardial function. Future work will analyze specific patient groups and assess other changes in velocity markers.

## Funding

AHA 12GRNT 12080032

